# Bottom-up approach synthesis of core-shell nanoscale zerovalent iron (CS-nZVI): Physicochemical and spectroscopic characterization with Cu(II) ions adsorption application

**DOI:** 10.1016/j.mex.2020.100976

**Published:** 2020-06-25

**Authors:** Adewumi Oluwasogo Dada, Folahan Amoo Adekola, Ezekiel Oluyemi Odebunmi, Fehintoluwa Elizabeth Dada, Olugbenga Solomon Bello, Adeniyi Sunday Ogunlaja

**Affiliations:** a Industrial Chemistry Programme, Department of Physical Sciences, Landmark University, PMB 1001, Omu Aran, Nigeria; b Department of Industrial Chemistry, University of Ilorin, PMB 1515, Nigeria; c Department of Chemistry, University of Ilorin, PMB 1515, Nigeria; d Directorate of University Wide Courses, Landmark University, PMB 1001, Omu Aran, Nigeria; e Department of Pure and Applied Chemistry, Ladoke Akintola University of Technology, PMB 4000 Ogbomoso, Oyo, Nigeria; f Department of Chemistry, Nelson Mandela Metropolitan University, P.O. Box 77000, Port Elizabeth 6031, South Africa

**Keywords:** Core shell nZVI, Characterization, Surface chemistry, Morphology, Remediation, Endocrine disruptive compounds, Isotherms, Kinetics, Statistical validity

## Abstract

Single pot system in chemical reduction via bottom-up approach was used for the synthesis of core shell nanoscale zerovalent iron (CS-nZVI). CS-nZVI was characterized by a combination of physicochemical and spectroscopic techniques. Data obtained showed BET surface area 20.8643 m^2^/g, t-Plot micropore volume 0.001895 cm^3^/g, BJH volume pores 0.115083 cm^3^/g, average pore width 186.9268 Å, average pore diameter 240.753 Å, PZC 5.24, and pH 6.80. Surface plasmon Resonance from UV-Vis spectrophotometer was observed at 340 nm. Surface morphology from SEM and TEM revealed a spherical cluster and chain-like nanostructure of size range 15.425 nm −97.566 nm. Energy Dispersive XRF revealed an elemental abundance of 96.05% core shell indicating the dominance of nZVI. EDX showed an intense peak of nZVI at 6.2 keV. FTIR data revealed the surface functional groups of Fe–O with characteristics peaks at 686.68 cm^−1^, 569.02 cm^−1^ and 434 cm^−1^. In a batch technique, effective adsorption of endocrine disruptive Cu(II) ions was operational parameters dependent. Isotherm and kinetics studies were validated by statistical models. The study revealed unique characteristics of CS-nZVI and its efficacy in waste water treatment.

## Highlights

• CS-nZVI was synthesized under anaerobic condition via bottom-up approach.
• Physicochemical characterization, Surface functionality and morphology revealed the unique properties of CS-nZVI.
• Effective adsorption was achieved in a batch technique while isotherm and kinetic models were statistical validated.

## Graphical abstract

**Figure:**
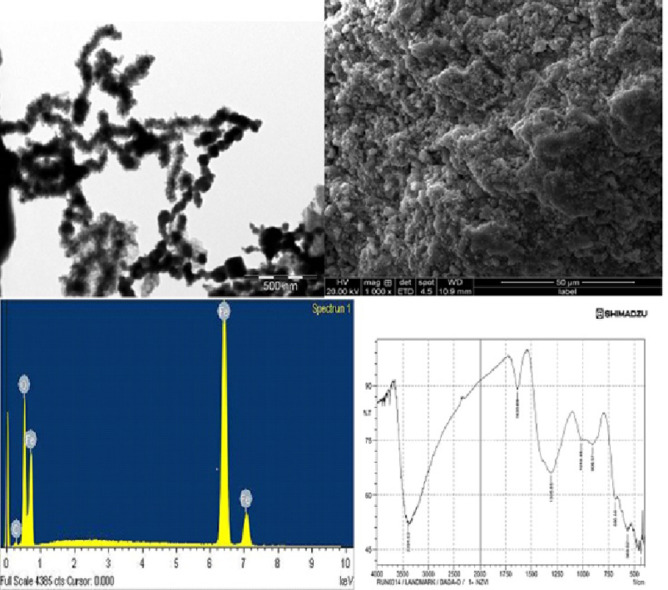

**Specifications Table**

**Table:** 

Subject Area:	Environmental Science
More specific subject area:	*Nanochemistry, Adsorption, Environmental Engineering*
Method name:	*Physicochemical and Spectroscopic Characterization of CoreshellnZVI with Application in Endocrine Disruptive Cu(II) ions Adsorption*
Name and reference of original method:	A.O. Dada, F.A. Adekola and E.O. Odebunmi. Kinetics and equilibrium models for Sorption of Cu(II) onto a Novel Manganese Nano-adsorbent. *Journal of Dispersion Science and Technology,* 37(1), (2016) 119 – 133. *DOI: 10.1080/01,932,691.2015.103461*
Resource availability:	*The data are available with this article*

## Method details

The field of nanotechnology is increasing in global relevance as current trend that has attracted several researchers based on its various applications. Development of nanomaterials with unique characteristics could be achieved via nanotechnology. Nanoparticles (NPs) are not simple molecules. They are materials containing different layers such as surface layers, shell layers and the core shell layers [1]. NPs have found relevance in environmental remediation. Building up of nanoparticles could be through top-down or bottom-up approach routes [2]. Based on physical and chemical properties, nanoparticles are classified into carbon-based [3]), metallic [4], ceramic, polymeric [5], semiconductor [6] and liquid-based [7]. Feasibility of size, facet and shape variation during synthesis gave metallic nanoparticles edge of various applications. Of all the members of metallic nanoparticles, zerovalent iron nanoparticles and their composite materials have shown distinctive applications in remediation [8], [9]. In addition, the sustainable synthetic route of iron nanoparticles and its compatibility for composite formation have also attracted interest of researchers. Zhu et al. [10] explored nanoscale zerovalent iron/nickel (GT-nZVI/Ni) prepared by green synthesis technology using green tea extracts and it was applied for uptake of Cr(VI) from groundwater. Biochar-supported nano zero-valent iron/nickel bimetallic composite (BC@nZVI/Ni) was synthesized using liquid phase reduction method and used to activate persulfate (PS) to degrade norfloxacin (NOR) in water [11]. Dada et al. [12] investigated the kinetics, mechanism, isotherm and thermodynamics studies of the liquid -phase adsorption of Pb(II) ions using wood activated carbon supported zerovalent iron nanocomposite (WAC-nZVI) synthesized by chemical reduction route using bottom-up approach. Bentonite Supported Nanoscale Zerovalent Iron Nanocomposite (B-nZVI) synthesized via chemical reduction using sodium borohydride was explored for uptake of Rhodamine B [13]. In this present study, the easy procedure for synthesis of Core Shell Nanoscale Zerovalent iron(CS-nZVI) via chemical reduction by bottom-up approach was explored. Proper priority and full consideration was given to analytical and spectroscopic characterization of CS-nZVI which were not fully explored by researchers. These were studied vis-à-vis Point of zero-charge (PZC), Brunauer-Emmett-Teller (BET), pore width and volume, Ultraviolet Visible (UV–VIS) spectrophotometer, Fourier Transform Infrared (FTIR), Scanning Electron Microscopy (SEM), Transmission Electron Microscope (TEM),Energy Dispersive X-ray (EDX) and X-ray Florescence (XRF). Application of CS-nZVI in sequestration of endocrine disruptive copper ions was also investigated. Pertinent adsorption conditions such as effects of initial copper ions concentration, contact time, pH, CS-nZVI dosage and ionic strength were studied. Statistical validity of kinetics and isotherm models which are seldomly reported was also examined. Mechanism and kinetic of the system were examined using Pseudo-first and second order and power function models. Isotherm and kinetic models were both validated by sum of square error (SSE), Chi-square test (χ^2^) and normalized standard deviation (Δq).

## Materials and methods

### Reagents

Analytical grade reagents majorly purchased from Sigma-Aldrich was used. Deionized deoxygenated water (sparged with nitrogen gas) and ordinary single distilled-deionized water was used all through the synthesis. Sodium Borohydride (NaBH_4_, CAS No.: 16,940–66–2) was used for the chemical reduction, other reagents were: FeCl_3_·6H^2^O (Sigma-Aldrich, USA,≥98%, CAS Number 10,025–77–1), absolute ethanol (BDH, CAS No.: 64–17–5) and HNO_3_ (Sigma-Aldrich, USA), Sodium Hydroxide (NaOH, CAS No.: 1310–73–2),CuSO_4_·5H^2^O (Breckland Scientific Batch No. 6688), were used without further purification. Vacuum filtration setup, 0.45 µm milipore filter paper

### **Synthesis** of core shell nano**scale zerovalent iron (CS-nZVI)**

**Fig. 1:**
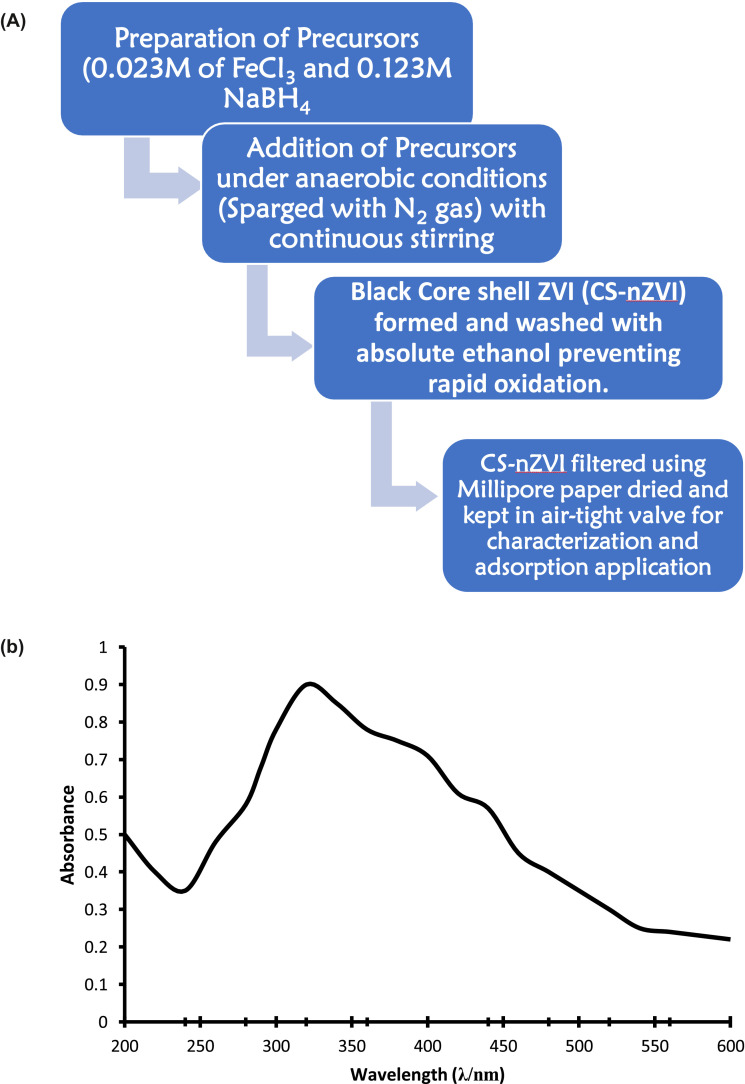
(A): Synthetic route of Core shell nanoscale zerovalent iron (CS-nZVI). (B): UV–Vis spectrum of CS-nZVI.

CS-nZVI was synthesized following the following route (Fig. 1a): solution A containing Ferric Chloride of 0.023 M was prepared by weighing calculated amount of 6.22 g of FeCl_3_·6H_2_O which was dissolved in a mixture of 1000 mL of absolute ethanol and deionized deoxygenated water (sparged with nitrogen gas) in ratio 1:4 respectively. Solution B of 0.125 M was also prepared by weighing 4.70 g of NaBH_4_ dissolved in 1000 mL of deionized deoxygenated water (sparged with nitrogen gas). Solution A was agitated on magnetic stirrer for 30 min before solution B was gradually added drop wisely at a flow rate of 2 drops per second. Under anaerobic environment, the ratio of volume of solution B to solution A was 2:1 and reaction performed nitrogen controlled glove box. As soon as NaBH_4_solution was added to Fe^3+^ solution, black solid appeared indicating the formation of zerovalent nanoparticles showing a magnetic property by sticking to the wall of magnetic stirrer. The mixture was further stirred for 3 hrs to give allowance for complete reduction reaction and evolution of hydrogen gas [8], [14]. Ferric ion was reduced to zerovalent iron nanoparticles under inert condition according to the reaction:

(1) $$4Fe^{3+}+3B{H_{4}}^{-}+9H_{2}O\to 4Fe^{0}↓+3H_{2}B{O_{3}}^{-}+12H^{+}+6H_{2}↑$$

The synthetic route is well illustrated in Fig. 1a

### Characterization of core shell nanoscale zerovalent iron (CS-nZVI)

#### Surface charge (Point of zero charge) and surface area determination

Point of zero charge was determined by pH variation from 2 to 12. This was carried out by adjustment with 0.1 M HNO_3_ or 0.1 M NaOH improving on the protocol in the literature [15]

Volume and size of pores of CS-nZVI with the surface area were determined through MicrometriticsAutoChem II Chemisorption Analyzer by BET and Barrett-Joyner-Halenda (BJH) methods.

#### Surface plasmon resonance and surface functional group by UV–VIS and FTIR

The Absorption band arising from the surface plasmon resonance in the CS-nZVI was measured using a double beam Beckmann Coulter DU 730 Life Science UV–Vis spectrophotometer (USA). The information on the molecular environment and surface functional group of CS-nZVI was gotten from the FTIR spectroscopy using Shimadzu FTIR model IR 8400S (Japan)

#### Surface morphology and elemental distribution by TEM, SEM-EDX and XRF

The dimension and size of CS-nZVI was determined employing the use of A Zeiss Libra 120 TEM at 80 kV(Germany). Surface morphology of CS-nZVI was analysed employing SEM integrated with EDX analyser while the spectra were obtained by TESCAN Vega TS 5136LM typically at 20 kV (Czech Republic) at a working distance of 20 mm. The determination of elemental constituents of the precursors and nanocomposites were carried out using MINILPAL 4 Energy Dispersive XRF.

## Batch adsorption process

### Preparation of copper stock solution

Through precise calculation of measured amount of 2.5 g CuSO_4_·5H_2_O dissolved in 1000 mL, 1000 ppm of Cu(II) ions stock solution was prepared. Lower concentrations (10 – 200 ppm) for the study was prepared by serial dilution

### Batch equilibrium and kinetics studies with statistical validity

Batch adsorption experiment was done by contacting and agitating CS-nZVI (100 mg adsorbent dose) with 50 mL Cu(II) ions (10 – 200 ppm) concentration. Residual concentrations after adsorption were determined by Atomic Absorption Spectrophotometer (AAS) model AA320N (Shanghai, China). Using Eqs. (2) and (3), adsorption capacity (Q_e_) of CS-nZVI and percentage removal efficiency (%RE) were evaluated [16]:

(2) $$Q_{e}=\frac{\left(C_{o}-C_{e}\right)V}{W}$$

(3) $$\%\;RE=\frac{C_{o}-C_{e}}{C_{o}}\times 100$$

Langmuir and Freundlich isotherm are the two most common models employed to describe the equilibrium data. In order to carry out kinetic studies, the adsorption kinetic experiments were conducted at optimum conditions for Cu^2+^ ions at contact time ranging from 10 – 120 min [4], [7]. The kinetic data were fitted into pseudo first-order and pseudo second-order models. Presented in Eq. (4) - 12 are isotherm, kinetics and statistical validity model equations relevant to this study. In effect of pH, test solution pH was determined using pH meter. Variation in pH was done using dilute solutions of hydrogen trioxonitrate (V) acid (0.1 M) and sodium hydroxide (0.1 M) solutions. The pH reading was recorded using a pH meter model (HANNA HI-2550 pH/ORP/ISE, EC/TDS/NaCl Benchtop Meter). Investigation on ionic strength was carried out by using sodium chloride solution of different concentrations (0.001 M, 0.01 M, 0.1 M, 0.5 M and 1.0 M)

### Data analysis and statistical validity

After batch equilibrium and kinetics studies, data obtained were fitted to the following models presented in Eq (4) −(12). Data from equilibrium adsorption are is usually model by isotherm which relates the relative concentration of solute adsorbed onto the solute in solution.

Adsorption isotherm models were explored in order to reveal the governing principle behind the binding of adsorbate to adsorbent at optimum operational parameters [17], [18]. In this study, equilibrium data were fitted to two most common isotherm models, Langmuir and Freundlich as shown in Eq. (4) – (6).

In order to understand the rate controlling steps and mechanism of the liquid-phase adsorption of Cu^2+^ onto CS-nZVI, the data obtained from contact time were fitted to another two most common models; Pseudo first-and-pseudo second-order models. Eqs. 7 – 8 show the linear expression of these kinetics models. The suitability, agreement and best fit among the kinetic and isotherm models are examined and judged not only with regression coefficient *(R^2^),* but also by sum of square error (SSE) (Eq. (10)), Chi-square test (χ^2^) (Eq. (11)) and Normalized standard deviation (Δq) (Eq. (12)).

## Isotherm models

### Langmuir

(4) $$C_{e}\;/\;q_{e}=\frac{1}{K_{L}q_{max}}+C_{e}\;/\;q_{max}$$

(5) $$R_{L}=\frac{1}{1+K_{L}C_{o}}$$

### Freundlich

(6) $$\ell ogq_{e}=\ell ogCe+\frac{1}{n}logK_{f}$$

### Kinetics models

Largergren Pseudo-first order

(7) $$log\left(q_{e}-q_{t}\right)=log\;q_{e}-\frac{k_{1}t}{2.303}$$

### Pseudo second-order

(8) $$\frac{t}{q_{t}}=\frac{1}{h_{2}}+\frac{t}{q_{e}}$$

(9) $$h_{2}=k_{2}q_{e}^{2}$$

### Statistical validity models

#### Sum of square error (SSE)

(10) $$SSE=\sum_{i=1}^{n}{\left(q_{e,cal}-q_{e,\exp}\right)}^{2}$$

#### Chi-square test (χ^2^)

(11) $$\chi^{2}=\sum_{i=1}^{n}\frac{{\left(q_{e,\exp}-q_{e,cal}\right)}^{2}}{q_{e,cal}}$$

#### Normalized standard deviation (Δq)

(12) $$\Delta q\left(\%\right)=100\frac{\sqrt{\sum_{i=1}^{n}{\left(\frac{q_{e,\exp}-q_{e,cal}}{q_{e,\exp}}\right)}^{2}}}{n-1}$$

## Results and discussion

### Physicochemical characterization of CS-nZVI

Table 1 shows the following physicochemical properties of CS-nZVI vis-à-vis surface area, micropore area, BJH Adsorption cumulative surface area of pores, pore volume, pore diameter, pore width, average particle size by Brunauer-Emmett-Teller (BET) and Barrett-Joyner-Halenda (BJH) methods, point of zero charge (PZC) were determined. The point of zero charge is of basic importance in surface science, environmental science and colloidal technology. The point of zero charge (PZC) of CS-nZVI determined as 5.24 which revealed that adsorption of Cu^2+^ would take place at a pH > pH (pzc). It is inferred that more active binding will be available as a result of deprotonation and low electrostatic repulsion [19], [20].

**Table 1:** Physicochemical parameters of Core-shell Nanoscale Zerovalent (CS-nZVI).

Physicochemical Parameters	CS–nZVI
**pH**	**6.80**
**PZC**	**5.24**
**BET Surface Area**	20.8643 m²/g
t-Plot Micropore Area	4.4140 m²/g
t-Plot External Surface Area	16.4503 m²/g
BJH Adsorption cumulative surface area of pores	19.120 m²/g
**Pore Volume**	
Single point adsorption total pore volume of pores	
less than 1103.482 Å diameter at P/Po = 0.982136052:	0.097502 cm³/g
t-Plot micropore volume:	0.001895 cm³/g
BJH Adsorption cumulative volume of pores	0.115083 cm³/g
**Pore Size**	
Adsorption average pore width (4 V/A by BET):	186.9268 Å
BJH Adsorption average pore diameter (4 V/A):	240.753 Å

### Spectroscopic and morphological characterization of coreshell nanoscale zerovalent iron (CS-nZVI)

#### UV–VIS analysis of CS-nZVI

The CS-nZVI was synthesized via chemical reduction under inert environment. A small aliquot was drawn from the reaction mixture and the spectrum was taken from a wavelength scan from 200 nm to 600 nm. Depicted in Fig. 1b is the UV–VIS spectrum of CS-nZVI. The absorption band arose as a result of surface plasmon resonance of CS-nZVI. Peaks were observed between 200 nm – 600 nm for CS-nZVI and the wavelength with the maximum absorbance was at 340 nm. In metal nanoparticles, the conduction band and valence bands lie very close to each other in which electrons move freely. These free electrons give rise to a surface plasma resonance absorption band, occurring due to collective oscillation of electrons in resonance with light wave. This was in accordance with the literature report [21].

#### Surface functional group determination

**Fig. 2:**
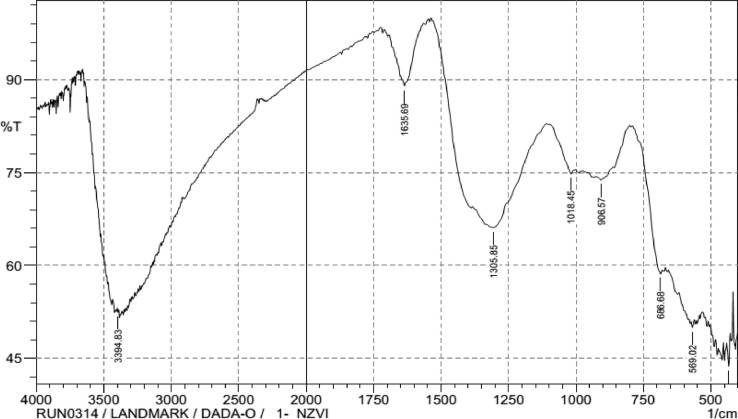
FTIR for Core shell Nanoscale Zerovalent Iron (CS-nZVI).

Fig. 2 showed the FTIR spectrum and characteristic frequencies denoting the functional group corresponding to vibration bands of CS-nZVI in the range of 400 – 4000 cm^−1^.The broad and intense peak around 3394 cm^−1^ correspond to the presence of O—H stretching from alcohol, 1632 cm^−1^ is attributed to H—O—H bending, the peak at1328 cm^−1^ corresponds to Cl. The remaining peaks at 686.68 cm^−1^, 569.02 cm^−1^, 434 cm^−1^ were attributed to Fe–O of the core shell zerovalent iron. This finding was supported by the report in the literature [22], [23]. The confirmation was provided by the XRF and EDX analyses of CS-nZVI. Table 2 gives the summary of the important FTIR bands of CS-nZVI with their possible functional groups and intensities of the CS-nZVI.

**Table 2:** Important FTIR bands of CS-nZVI with their possible functional groups.

Functional Groups	Vibration Bands/Peaks (cm^−1^)	Intensity
O-H stretching	3394.83	52.461
H—O—H bending	1635.69	88.99
- Cl	1305.85	66.122
	686.68	58.666
Fe–O stretching	569.02	49.975
	434	43.742

### Morphology determination by SEM and TEM

#### Transmission electron microscopy (TEM) analysis of CS-nZVI

**Fig. 3:**
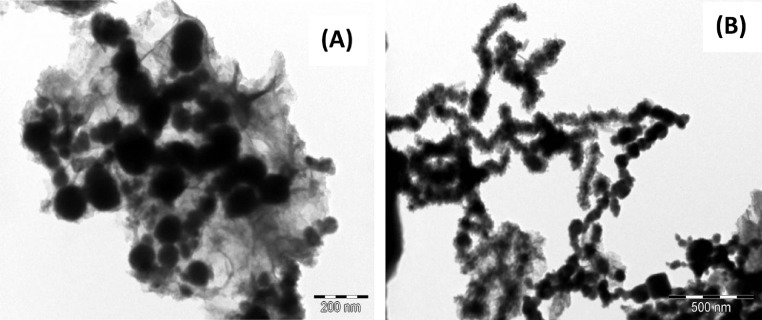
(A): TEM of CS-nZVI. (B): TEM of CS-nZVI.

Fig. 3 (a&b) showed the TEM micrographs of CS-nZVI. It is obvious that particles are spherical, snake-like and chain-like because of the magnetic nature in core shell zerovalent iron (Fe^0^) nanoparticles. This is supported by study in the literature where zerovalent iron nanocomposite was synthesized [24]. The size ranges from 15.425 nm – 97.566 nm [25].

**Fig. 4:**
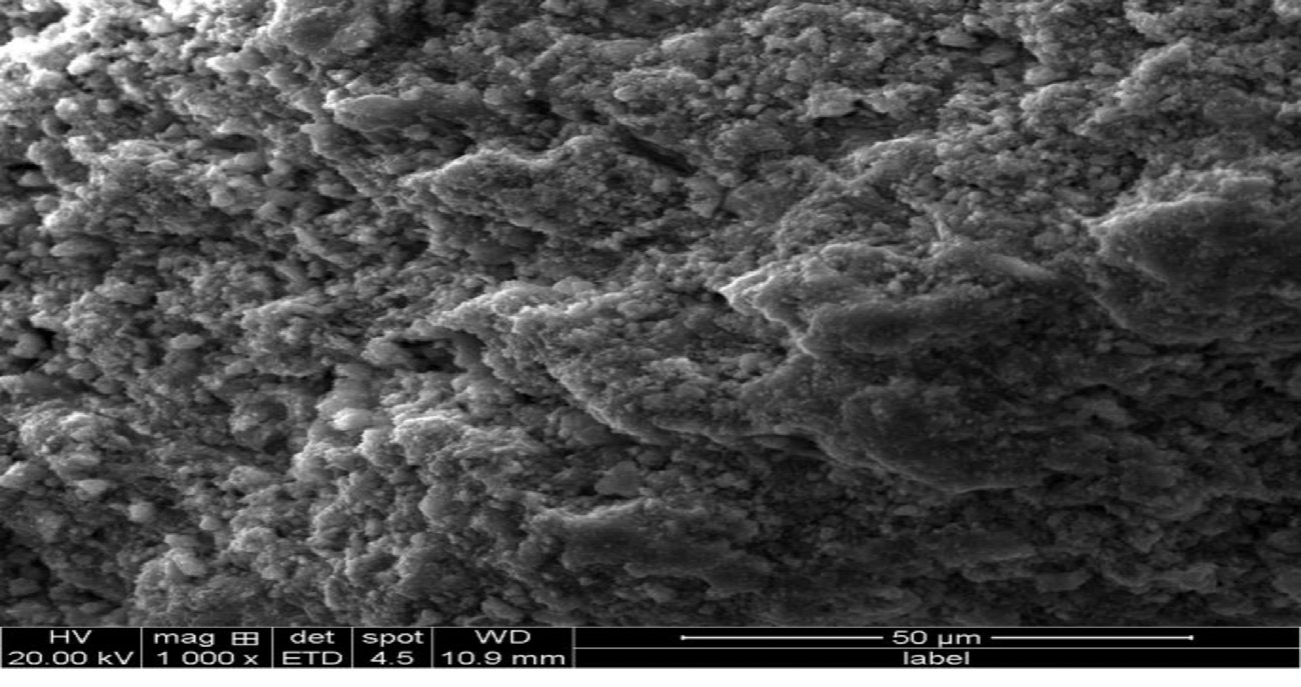
SEM micrograph of CS-nZVI.

Fig. 4 reveals the SEM micrograph of CS-nZVI. A spherical surface of chain-like, aggregated molecules is seen. The chain-like aggregation is an indication of its magnetic property. This spherical CS-nZVI particle provides a large surface area for the adsorption of toxicant and this is in agreement with the report in literature [14], [26].

### Determination of elemental and atomic constituents of CS-nZVI by XRF and EDX

#### X-ray florescence analysis of CS-nZVI

The chemical constituents of the adsorbents used for the sorption studies were determined using MINIL PAL 4 Energy Dispersive X-ray Florescence spectrometer. The elemental composition of nanoscale zerovalent iron (CS-nZVI) as revealed by X-ray Florescence (XRF) is presented in Table 3. CS-nZVI was prepared by chemical reduction of FeCl_3_ by NaBH_4_ in a single pot system by bottom-up approach. The element with the highest abundance of 96.05% is the core shell CS-nZVI. Its highest percentage is an indication of its dominance in the compound. Other compounds such as Al_2_O_3_ (0.70%), SiO_2_ (0.72%), P_2_O_5_ (0.37%), V_2_O_5_ (0.084%), CaO (0.13%), Cr_2_O_3_ (0.031%), MnO (0.043%), NiO (0.02%), ZnO (0.083%), Ag_2_O (1.2%), La_2_O_3_ (0.04%) and lost of mass on ignition (LOI) of 0.529 were present in trace amount.

**Table 3:** XRF analysis of Core shell Nanoscale Zerovalent Iron (CS-nZVI).

Compound	Wt (Unit)%
Fe_2_O_3_ (CS-nZVI)	96.05
Al_2_O_3_	0.70
SiO_2_	0.72
P_2_O_5_	0.37
CaO	0.13
V_2_O_5_	0.084
Cr_2_O_3_	0.031
MnO	0.043
NiO	0.02
ZnO	0.083
Ag_2_O	1.200
La_2_O_3_	0.04
LOI	0.529
Total	99.471

#### EDX analysis of CS-nZVI

**Fig 5:**
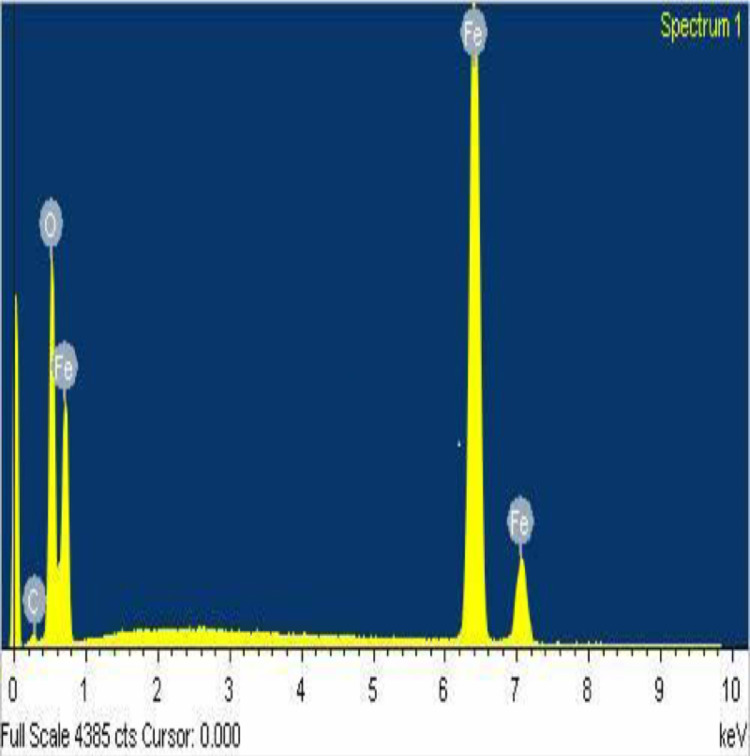
EDX spectrum of CS-nZVI.

Atomic and elemental distribution of CS-nZVI were revealed in EDX spectrum as depicted in Fig. 5. EDX analysis gives qualitative as well as quantitative status of elements that may be involved in formation of nanoparticles. It gives information on the elemental composition of the sample of interest and their relative abundance [27]. Presented in Fig. 5 and Table 4 are information from the EDX analysis of CS-nZVI. The intense peak of core shell iron nanoparticle is found at 6.4 eV [28]. It is obvious that core shell nanoparticles occupied 86.47% by weight indicating its dominance. Other elements present could be traceable to the additives during the course of the analysis. A confirmation of the nanoparticles and their composite was well provided from the EDX result which was supported by the XRF with reference to Table 3.

**Table 4:** EDX major Elemental percentage composition of CS–nZVI.

Element	Weight%	Atomic%
C K	1.72	4.75
O K	21.24	43.97
Fe K	86.47	51.28
Totals	109.43	100

#### Effect of adsorption operational parameters

**Fig. 6:**
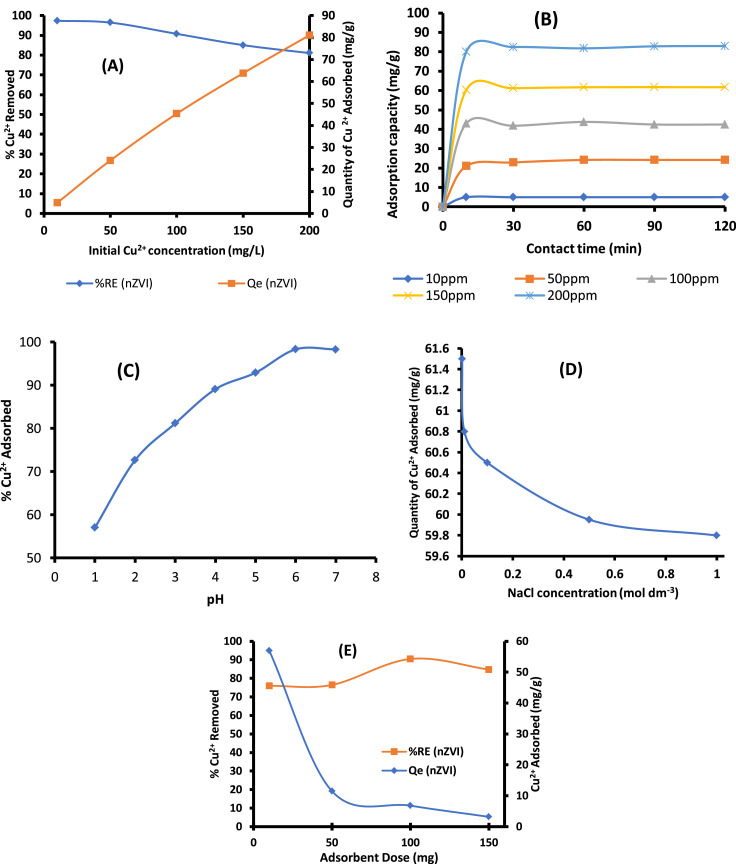
(A): Effect of initial Concentration on adsorption of ED-ions Cu(II) onto CS-nZVI. Experimental conditions:Vol of Cu^2+^solution = 50 mL; mg/LAdsorbent dose = 100 mg;pH = 6, contact time = 60 min and temperature = 25± 2 °C. (B): Effect of contact time on adsorption of ED-Cu(II) onto CS-nZVI. Experimental conditions:Cu^2+^Concentration=10 – 200 mg L^-1^; vol of Cu^2+^solution = 50 mL;Adsorbent dose= 100 mg; pH = 6, and temperature = 25± 2 °C. (C): Effect of pH on adsorption of ED-Cu(II)ions onto ions CS-nZVI. Experimental conditions: Vol of Cu^2+^solution = 50 mL; Adsorbent dose = 100 mg;pH = 6, contact time = 60 min and temperature = 25± 2 °C, Stirring speed = 200 rpm. (D): Effect of ionic strength on adsorption of ED-Cu(II) ions onto CS-nZVI. Experimental conditions:Cu^2+^Concentration=10 – 200 mg/L; Vol of Cu^2+^solution = 50 mL;Adsorbent dose= 100 mg; pH = 6, and temperature = 25± 2 °C, Stirring speed =200 rpm. (E): Effect of adsorbent dose on adsorption of ED-Cu(II) ion on CS-nZVI. Experimental conditions: Cu^2+^ Concentration= 150 mg/L; Vol of Cu^2+^solution = 50 mL; pH = 6, Stirring speed = 200 rpm, Contact time = 60 min, and Temperature = 25± 2 °C.

Studies have shown that effective adsorption depend on operational parameters that influence the chemistry of the process. Adsorption of endocrine disruptive Cu(II) ions was a function of effect of pH, ionic strength, initial Cu(II) ions concentration and contact time. Fig. 6 (A–E) shows the results of these operational parameters.

Effect of initial concentration plays a major role in the uptake of Cu^2+^ as a driving force to overcome the mass transfer resistance between the Cu^2+^-CS-nZVI system. The effect of initial concentration studied from 10 mgL^−1^ to 200 mgL^−1^ is presented in Fig. 6A. It was observed that at lower concentrations, the percentage removal efficiency increased because of the availability of more active sites until the adsorption sites were saturated at higher concentration between 150 mgL^−1^ and 200 mgL^−1^. The increase in adsorption capacity with an increase in initial Cu^2+^ concentration from 10 to 200 mgL^−1^ was as a result of the increase in driving force due to the concentration gradient developed between the bulk Cu^2+^ solution and surface of the nano-adsorbents [25]. At higher Cu^2+^ concentrations, the active sites of the nano-adsorbents were surrounded with Cu^2+^ and this continued until equilibrium was reached between 150 mgL^−1^ and 200 mgL^−1^.

Effect of contact controls the build-up of charges at solid-liquid interfaces [7], [30]. Displayed in Fig. 6B is the effect of contact time. The rate of reaction was rapid from 10 min until an optimum contact time was observed at 60 min after which a steady state approximation set in and a quasi-equilibrium situation was attained. A rapid contact time observed indicated a fast transport of metal ions from the bulk to the outer and inner surface of the nano-composite material. The adsorption uptake of Cu^2+^ at equilibrium increased from 4.96 mgg^−1^ to 82.82 mgg^−1^ as the initial Cu^2+^ concentration increased from 10 to 200 mgL^−1^

Considering Fig. 6C, at low pH values, the H^+^ concentration was high, which competed with copper cations for the same adsorption sites. However, at a higher pH values, deprotonation occurred, more sites were made available for the binding of Cu^2+^ to the surface of the adsorbent thereby the removal efficiency increased until equilibrium was reached after where no significant increase was noticeable [4], [31]

Effect of ionic strength was carried out because water system is always polluted with heavy metal ions together with other alkali or alkaline earth metal ions [25], [32]. These ionic strength increases the salinity and background electrolyte of the water body. Fig. 6D showed the influence of ionic strength on Cu^2+^ adsorbed onto the CS-nZVI at optimum conditions. It was observed generally from Fig. 6D that increase in ionic strength rapidly led to decrease in the percentage removal efficiency. Analysis of this plot showed a decrease in percentage of Cu^2+^ removed from 81.99% - 79.73% with reduction in quantity adsorbed from 61.49 mgg^−1^ – 59.79 mgg^−1^. The slight decrease is suggested to be due to possible formation of chloride complexes of Cu(II) with increase in chloride concentration, buffering capacities of the ions and unprecedented competition [32], [32]. Advantageously, percentage removal efficiency of 79.73% indicated that CS-nZVI would be an effective adsorbent in treatment of waste water polluted with different electrolytes.

Analysis of Fig. 6E showed that percentage of Cu^2+^ removed increased with an increase in the adsorbent dose as a result of increase in the number of active binding exchangeable sites. Fig. 6E demonstrated that the removal efficiency of Cu^2+^onto CS-nZVI increased from 75.96% – 90.47% with increase in adsorbent dose from 10 mg – 100 mg. No significant adsorption was observed beyond 100 mg as the surface was saturated and the residual concentration was extremely low. This finding is supported by reports in the literature [34].

### Batch adsorption isotherm studies with statistical validity

**Figs. 7:**
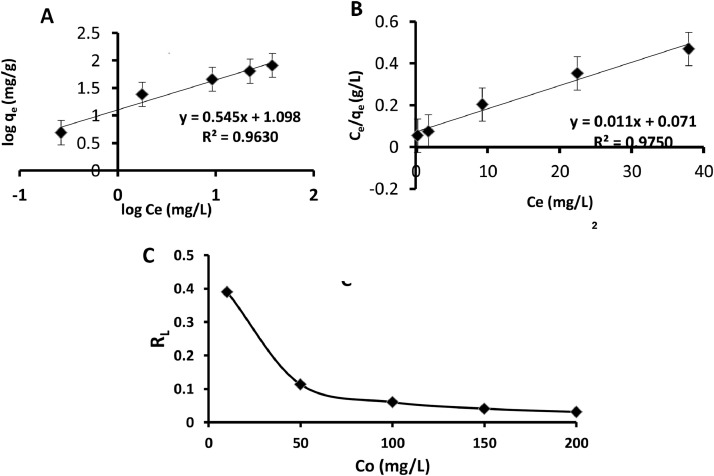
(A-B): Linear plots of (A) Freundlich (B) Langmuir for sorption of Cu^2+^ onto CS-nZVI. (C): Langmuir dimensionless separation factor for sorption of Cu^2+^ onto CS-nZVI.

Revealed in isotherm models is the information about the interaction between adsorbate-adsorbent system. In this study, equilibrium data were fitted to two most common isotherm models: Langmuir and Freundlich (Fig. 7A &7B respectively). Statistical validity was introduced to validate the isotherm models. This is not common in most research where isotherm models are used in describing the equilibrium data. Employed to validate these isotherm models are sum of square error (SSE), Chi-square (χ^2^), and normalized standard deviation (∆q). Inscribed on each plot as displayed in Fig. 7(A–B), are error bars. Fig. 7C is the plot of the Langmuir dimensionless, separation factor. The value presented on Table 5 lying between 0.390 and 0.0309 indicated that the sorption of Cu^2+^ onto CS-nZVI was favourable. The values of the Freundlich isotherm constants, *K_f_* and *n_f_* which indicated the sorption capacity and intensity were respectively evaluated to be 12.5401 and 1.8325 (Table 5). These parameters are characteristic of the adsorbent-adsorbate (CS-nZVI-Cu^2+^) system [35], [36]. The Langmuir isotherm suggested that adsorption took place only at specific localized sites on the surface of CS-nZVI where Cu^2+^ saturated coverage corresponded to complete occupancy of these sites; each CS-nZVI site could accommodate unity value of Cu^2+;^ due to the homogeneous nature of the CS-nZVI surface, there was no interaction between Cu^2+^adsorbed on different surfaces of CS-nZVI hence there was no phase transition. The maximum monolayer coverage capacity (*q_max_*) evaluated was 99.09 mgg^−1^. This is much higher when compared with other adsorbents such as Fe_3_O_4_ magnetic nanoparticles coated with humic acid (46.3 mgg^−1^) [37]; Magnetic gamma-Fe_2_O_3_ nanoparticles coated with poly-l-cysteine (42.9 mgg^−1^) [38]; Amino-functionalized magnetic nanosorbent (25.77 mgg^−1^) [39]. Langmuir isotherm constant (Lmg^−1^), *K_L_* related to the energy of adsorption was evaluated to be 0.1563. The essential feature of the Langmuir isotherm model expressed in terms of the dimensionless constant, R_L_ (Fig. 7C) also known as the separation factor with the values less than unity (*R_L_*=0.3591, Table 5) is an indication of the favourability of the sorption of Cu^2+^ onto CS-nZVI. The equilibrium data were better described by Langmuir isotherm model judging from the R^2^ 0.9752 with relatively low values of SSE, χ^2^, ∆q. [4], [29]. Hence, the adsorption of EDC—Cu^2+^ is chemisorption in nature

**Table 5:** Isotherm models parameters for the sorption of Cu^2+^ onto CS-nZVI.

Freundlich	Parameters	Langmuir	Parameters
*k_F_*	12.5401	*Q_max_ (mgg^−1^)*	90.0901
*1/n_F_*	0.5457	*K_L_ (Lmg^−1^)*	0.1563
*n_F_*	1.8325	*R_L_*	0.3591
*R^2^*	0.963	*R^2^*	0.9752
*q_e_, exp*	63.78	*q_e_, exp*	63.78
*q_e_, cal*	68.4783	*q_e_, cal*	70.1066
*SSE*	22.074	*SSE*	40.0259
*X^2^*	0.3224	*X^2^*	0.5709
*Δq*	1.8416	*Δq*	2.4799

### Batch adsorption kinetics studies with statistical validity

**Figs. 8:**
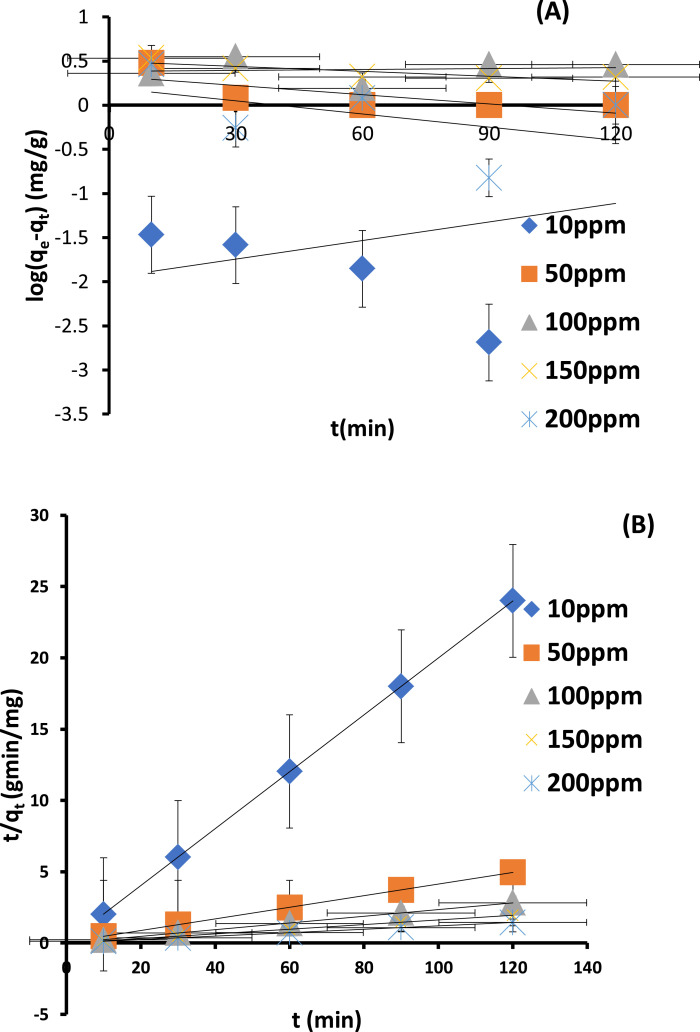
(A-B): Linear plots of (A) Pseudo first-order (B) Pseudo second-order kinetics model at various concentrations for adsorption of Cu^2+^ onto CS-nZVI.

Employed to investigate the mechanism and the rate determining step are the two most common kinetic models in adsorption studies: pseudo first-order (Eq. (7)) and pseudo second-order (Eq. (8)). The major reason why statistical validity models were used is that recent studies have shown that regression coefficient (*R^2^*) alone may not be fully sufficient to determine and judge the model that would best describe the mechanism and rate determining step. Hence, the kinetics models have been validated using sum of square error, chi-square test and normalized standard deviation. Presented in Figs 8 (A-B) are pseudo first-order model and pseudo second-order models. Error bars on each of the plots were indications that statistical validity models were employed to validate the best kinetic model. Evaluated parameters shown in Table 6 revealed that pseudo-second order best described the mechanism of the system across all the concentrations.

**Table 6:** Kinetics parameters for the sorption of Cu^2+^ onto CS-nZVI at various initial Cu^2+^ concentrations.

Initial Concentration					
Kinetics Model parameters	10 ppm	50 ppm	100 ppm	150 ppm	200 ppm
***Pseudo first-order***					
*q_e_,exp (mg/g)*	4.9859	24.1981	43.8152	61.684	81.77
*q_e_, cal (mg/g)*	0.01109	2.1311	2.3147	3.1261	1.5816
*k_1_(*min*^−1^)*	−0.0161	8.065 × 10^−3^	−9.212 × 10^−4^	4.375 × 10^−3^	1.152 × 10^−2^
*h_1_ (mg/g/*min*)*	1.785 × 10^−4^	1.717 × 10^−2^	2.22 × 10^−3^	1.367 × 10^−2^	1.822 × 10^−2^
*R^2^*	0.1029	0.5549	0.0147	0.7275	0.2127
*SSE*	24.7487	486.952	1722.292	3429.027	6430.179
*χ^2^*	2231.626	228.498	744.0668	1096.902	4065.617
*∆q*	24.94439	22.79828	23.67928	23.73302	24.51645
***Pseudo Second-order***					
*q_e_, exp (mg/g)*	4.9859	24.1981	43.8152	61.684	81.77
*q_e_, cal (mg/g)*	5.005	24.6305	42.5532	61.728	83.333
*k_2_(g/mg/*min*)*	1.3441	0.02813	3.7301	3.8572	2
*h_2_ (mg/g/*min*)*	33.67	17.0648	158.73	238.095	166.667
*R^2^*	1	0.9999	0.9999	1	0.9999
*SSE*	3.648 × 10^−4^	1.869 × 10^−1^	1.5926	1.936 × 10^−3^	2.4429
*χ^2^*	7.288 × 10^−5^	7.591 × 10^−3^	3.743 × 10^−2^	3.136 × 10^−5^	2.932 × 10^−2^
*∆q*	0.09577	0.44673	0.72007	0.01783	0.47786

The higher adsorption rate (*h_2_*) from pseudo-second order was an indication of a fast adsorption process. The experimental quantity adsorbed (q_e_, exp) and calculated quantity adsorbed are in good agreement in pseudo second-order parameters but far part in pseudo first-order. The regression coefficient (*R^2^*) in pseudo second-order parameters are very close to unity and in some cases, it is exactly unity while in pseudo first-order, the R^2^ values were very low across the concentrations. Previous studies carried out showed that the better the agreement between the q_e_, *exp* and q_e_, *cal,* the lower the values of these statistical tools, the better the model [7], [40]. From the analysis of the statistical validity model, the low values of SSE, χ^2^ and ∆q (Table 6) further supported that pseudo second-order model best described the kinetic and mechanism of the adsorption process. The kinetic and mechanism of adsorption of EDC—Cu^2+^ was better described by pseudo second-order model supporting chemisorption adsorption

## Conclusion

Synthesis of core shell zerovalent iron nanoparticles (CS-nZVI) was achieved using bottom-up approach in a single pot system via chemical reduction. CS-nZVI was characterized by physicochemical and spectroscopic techniques. The distinct physiochemical properties of pH (pzc) 6.80, BET surface area 20.8643 m²/g, pore characteristics in terms of the width (186.9268 Å) and BJH adsorption average pore diameter 240.753 Å support its suitability for adsorption studies. Absorption band of CS-nZVI was observed at 340 nm. Atomic abundance by weight was 96.05% and intense peak of zerovalent iron from EDX micrograph was observed between 6.4 eV and 7.1 eV with atomic weight of 86.47%. TEM and SEM micrographs revealed a spherical and chain-like structure as a result of magnetic force in CS-nZVI. Adsorption of endocrine disruptive copper ions was attained at optimum conditions. Advantageously, 81.99% removal efficiency was attained in the presence of co-existing ions and background electrolyte in effect of ionic strength. Equilibrium data were described by Freundlich and Langmuir isotherm models with R^2^>0.96. Based on *R^2^* value, sum of square error (SSE), chi-square test (χ^2^) and normalized standard deviation (Δq), the kinetics and mechanism of the system were better described by pseudo second-order models. This study shows the ease of synthesis of CS-nZVI. Its unique characteristic properties confirm that CS-nZVI is applicable in adsorption of endocrine disruptive heavy metal ion.
